# Metachronous Bilateral Painless Foot Drop Secondary to Spinal Stenosis: Case Report

**DOI:** 10.7759/cureus.2276

**Published:** 2018-03-05

**Authors:** Luis A Robles

**Affiliations:** 1 Section of Neurosurgery, Hospital Cmq

**Keywords:** foot drop, painless foot drop, bilateral foot drop, l5 radiculopathy, spinal stenosis, lumbar degenerative disease

## Abstract

Foot drop (FD) is an uncommon manifestation of nerve root compression secondary to lumbar degenerative disease (LDD). In most of these patients, FD is unilateral and is associated with low back pain and leg pain. A small number of cases have been described with bilateral FD, which is reported to occur simultaneously in a synchronous fashion. A 63-year-old male with a remote history of spinal surgery due to left painless FD presented with a new episode of right FD, this new FD initiated suddenly and it was not associated with low back pain or radicular pain. Magnetic resonance imaging (MRI) showed bilateral lumbar stenosis at L3-4 and subarticular stenosis at right L4-5 level causing compression of right L5 nerve root. Fenestrations, removal of flavum ligament and medial facetectomy were performed bilaterally at L3-4 and on the right side at L4-5 level. Postoperatively, the patient experienced progressive improvement on right foot dorsiflexion. The occurrence of bilateral painless FD presenting in a metachronous way as observed in the present case is a very unusual scenario. To the best of author’s knowledge, cases with these characteristics have not been previously reported.

## Introduction

Foot drop (FD) is characterized by the inability or difficulty in moving the foot upwards (dorsiflexion). Most of the time, FD is caused by a neurological affection. The source of the neurological impairment can be central (parasagittal cortical area, spinal cord) [1] or peripheral (neuropathies of the deep peroneal, common peroneal, sciatic nerve, lumbar plexus neuropathy or lumbar radiculopathy). Very rarely, FD may be caused by injury to foot dorsiflexors secondary to direct trauma or compartment syndromes.

Low back pain and sciatica are the most common manifestations of lumbar degenerative disease (LDD). Nonetheless, the presence of FD is an uncommon sign that can be part of the clinical findings in cases of LDD. In these patients, FD usually is unilateral and almost always is associated with radicular pain. It is reported in the literature that the incidence of FD in cases of LDD is approximately 8% [2,3]. A recent statistical analysis study showed that the risk of FD in cases of lumbar disc herniation is higher in cases of diabetes, lateral recess or foraminal herniation, disc calcification and canal occupancy greater than 50% [4].

The use of the term "metachronous" in medicine refers to the occurrence of a similar second event at a different time, and normally is used in oncology to describe the presence of new tumors.

This paper presents an unusual case of bilateral painless FD that occurred metachronously in the same patient, as a manifestation of lumbar spinal stenosis. Such situation has not been previously reported.

## Case presentation

This is a case of 63-year-old male. He had a history of left L4-5 lumbar spine surgery. He referred no history of diabetes, hypertension or peripheral vascular disease. At that time, he experienced progressive painless foot drop with no other associated symptoms. Although the surgery was performed with a delay of eight months, he achieved complete improvement after the procedure.

Eight years later, the patient suffered a sudden onset of right FD which started three weeks before being initially assessed, he denied any low back or leg pain. The patient referred also occasional claudication while he was walking.

**Figure 1:**
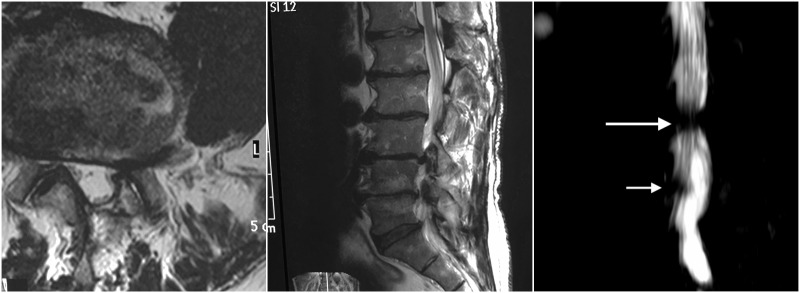
Lumbar spine magnetic resonance imaging (MRI) showing spinal stenosis. Axial MR image at L4-5 on T2-weighted imaging. Subarticular stenosis on right side is observed. Note partial laminar defect on left side, secondary to previous surgery in that area. Left subarticular stenosis is also observed. Center. Right parasagittal MR on T2-weighted imaging. Stenosis is observed at L3-4 and L4-5 segments. Modic changes are present mainly at L4-L5. Right. MR myelography. Bilateral stenosis is observed at L3-4 (large arrow), right stenosis at L4-5 is also demonstrated compressing the right L5 nerve root (small arrow).

Neurological examination on the left leg was normal. Examination of right leg revealed a numb area in the dorsal foot and big toe. Dorsiflexors of foot showed a score of 0 on manual muscle testing. In addition, he had marked weakness of inversion and eversion of the foot; extensor hallucis longus and gluteus medius muscles were also weak. His plantar flexion was normal. Myotatic reflexes were symmetrical. The straight leg raising test was negative. There was no low back tenderness. Figure 1 displays a magnetic resonance imaging (MRI) showing bilateral stenosis at L3-4 level, and lateral stenosis at the right side of L4-5. Furthermore, Type I Modic changes were also observed. Lumbosacral X-ray films only showed findings of spondylosis.

The patient underwent to bilateral L3-4 fenestrations and medial facetectomy, the same procedure was performed at right L4-5 level. Facet and flavum ligamentum hypertrophy were observed during surgery. After surgery, the patient was sent to rehabilitation. Four months later, foot dorsiflexion improved completely.

## Discussion

FD is usually caused by lower motor neuron pathology. When a patient presents with FD associated with sciatica or low back pain, the source of pathologic area pinpoints to the lumbar spine. Pain is the outstanding presenting symptom in patients with lumbar nerve root compression caused by LDD; for this reason, it is difficult to explain why some patients show muscle weakness as the only manifestation of the same problem. In cases of painless FD, the most common cause is peripheral neuropathy and less frequently a central nervous system dysfunction. In the latter scenario, FD very likely will be associated with another upper neuron motor clinical findings [1].

When FD is caused by a lower motor lesion, the first step is differentiating between a peripheral neuropathy and radiculopathy secondary to spinal pathology. L5 radiculopathy is the most common described cause of FD related to LDD. Although the L4/5 level is the most frequent involved spinal segment causing FD, abnormalities at L3-4 and L5-S1 have been also implicated causing FD [5]. The common peroneal nerve, which is the terminal continuation of the lateral trunk of the sciatic nerve, predominantly consists of fibers from the L5 nerve root, though the intermixture of fibers from the L4 and S1 roots is common. Via the peroneal nerve, L5 provides innervation to ankle dorsiflexors (tibialis anterior, extensor hallucis longus, and extensor digitorum longus muscles) and foot evertors (peroneus longus and brevis muscles). L5 also supplies the tibial nerve, which innervates foot inversors. Therefore, with an L5 lesion, the patients often exhibit, in addition to weakness of dorsiflexion of the foot, paresis of foot inversion and eversion [6]. When FD is caused by a common peroneal nerve lesion, this is associated with paresis on eversion, but foot inversion is normal. In these cases, electromyography and nerve conduction studies may locate the level of lesion and also can establish the pathophysiology and prognosis of the disorder. In the current case, FD was associated with foot inversion and eversion weakness which indicates an L5 nerve root disturbance, probably having L4 nerve root affection secondary to stenosis of L3-4 as an additional factor.

A literature search was performed in order to review the characteristics of low back pain and radicular pain, as well as the presence of uni- or bilateral involvement, in cases of FD related to LDD. Special attention was paid to articles describing this clinical information.

FD related to LDD is almost always accompanied by low back pain or radicular pain. The series with the highest number of patients with FD related to LDD reports that 100% of 135 patients suffered low back pain and leg pain before FD [3]. Girardi, et al. [7] report 55 patients with FD, and all of them experienced associated radicular pain. Despite the very high incidence of radicular pain in cases of FD related to LDD, there are a few reports describing the presence of painless FD. Garrido and Rossenwaser report two cases of patients with unilateral painless FD secondary to L5 nerve root compression caused by L4-5 disc herniation [6]. A case series by Aono, et al. [8] report 46 patients with FD related to LDD, of whom 75% suffered radicular pain, and 17% of them presented with painless FD. In another paper by Aono, et al. [9], they report the highest amount of patients with painless FD related to LDD. They described 20 patients with painless FD, of which only one had bilateral FD. It is worth mentioning that seven of these patients had a history of radicular pain before presenting with painless FD.

Bilateral FD caused by LDD is rarely reported in the literature. Those documented cases have been synchronous in their presentation in that they were found during the same examination [3,8,10]. Kun, et al. report 18 patients with synchronous bilateral drop foot, and some of them showed asymmetry in the severity of weakness on both sides [3].

According to the previous information, most patients with FD related to LDD present with associated radicular pain. When a patient experiences bilateral FD, normally this occurs synchronously. So, observing a patient with metachronous bilateral painless FD is a unique event.

In the current case, the past history of left FD secondary to LDD coupled with the clinical findings observed on examination suggested that probably the lumbar spine was the problematic area in this patient.

## Conclusions

The presence of painless FD secondary to LDD is very rare, and even rarer is the occurrence of this condition in a bilateral and metachronous presentation. Although this is an exceptional event, physicians should be aware of this clinical scenario, especially when the patient has a history of the same pathology on the contralateral side.
